# Reduced Epithelial CD1a+ Langerhans Cell Density Is Associated with Advanced Stage and Nodal Positivity in Head and Neck Squamous Cell Carcinoma: A Cross-Sectional Immunohistochemical Study

**DOI:** 10.3390/ph19101564

**Published:** 2026-10-01

**Authors:** Cristina Stefania Dumitru, Raul Patrascu, Marius Liviu Moise

**Affiliations:** 1 Department II of Microscopic Morphology, Discipline of Histology, “Victor Babes” University of Medicine and Pharmacy Timisoara, E. Murgu Square, No. 2, 300041 Timisoara, Romania; cristina-stefania.dumitru@umft.ro; 2 Department of Functional Sciences, “Victor Babes” University of Medicine and Pharmacy Timisoara, E. Murgu Square, No. 2, 300041 Timisoara, Romania; 3 Radiology Department, Emerald Medical Center, Corneliu Coposu Boulevard 14, 300552 Timisoara, Romania; moiseliviumarius@gmail.com

**Keywords:** head and neck squamous cell carcinoma, Langerhans cells, chronic inflammation, tumor microenvironment, vascular endothelial growth factor, lymphangiogenesis, E-cadherin, immune evasion, biomarker, nodal metastasis

## Abstract

**Background/Objectives**: Chronic inflammation is increasingly recognized as a driver of head and neck squamous cell carcinoma (HNSCC) progression, but the interplay between local immune surveillance and angiolymphatic remodeling remains incompletely characterized. We hypothesized that advanced HNSCC stage would be associated with lower epithelial CD1a+ Langerhans cell density alongside higher proliferation, reduced E-cadherin-mediated adhesion, and higher angiogenic/lymphangiogenic activity, and aimed to relate this marker pattern to tumor stage defined by combined histopathological and radiological criteria. **Methods**: Fifty HNSCC specimens were included (mean age, 63.9 ± 8.7 years; 86% male; 88% current or former smokers). Specimens were evaluated immunohistochemically for CD1a (primary Langerhans cell metric) and S100 (supportive marker), Ki-67, E-cadherin, VEGF, and Ki-67/D2-40 double staining (proliferating lymphatic vessel density (pLVD), reported descriptively only), alongside hematoxylin and eosin (H&E)-based histological grading and combined histopathological/radiological (CT/MRI) staging. Associations were assessed using Spearman correlation, Mann–Whitney U/chi-square tests, and multivariable logistic regression. **Results**: CD1a+ epithelial density, the primary Langerhans-cell metric, was significantly lower in advanced-stage and node-positive tumors (median 22.30 vs. 32.75 cells/mm^2^, *p* = 0.0008; and 19.05 vs. 31.15 cells/mm^2^, *p* < 0.0001, respectively), while the Ki-67 index and VEGF immunoreactive score were correspondingly higher (all *p* ≤ 0.001), with a parallel decrease in E-cadherin H-score. CD1a+ density showed moderate inverse or direct correlations with the other evaluated markers (|ρ| = 0.56–0.64; all *p* < 0.001). On multivariable analysis, lower CD1a+ epithelial density remained associated with nodal disease (pathological or clinical/radiological nodal status) after adjustment for T category (OR = 5.98 per 10-cell/mm^2^ decrease; 95% CI, 1.75–20.35; *p* = 0.004), together with T category (OR = 12.07; 95% CI, 1.99–73.06; *p* = 0.007), a finding robust to marker specification in a sensitivity analysis using the S100/CD1a composite metric (OR = 5.55; 95% CI, 1.61–19.18; *p* = 0.007). **Conclusions**: In this cross-sectional cohort, lower epithelial CD1a+ Langerhans cell density was associated with advanced stage and nodal positivity and occurred alongside higher proliferative activity, higher VEGF expression, and lower E-cadherin expression. These routinely assessable tissue biomarkers may provide a feasible framework for future biomarker-based risk stratification and warrant investigation for potential diagnostic adjunctive value. However, prospective validation in independent cohorts, including formal assessment of diagnostic performance, is required before any diagnostic, prognostic, predictive, or treatment-selection role can be proposed.

## 1. Introduction

Head and neck squamous cell carcinoma (HNSCC) remains one of the most common malignancies worldwide, accounting for approximately 890,000 new cases and 450,000 deaths annually, with persistently poor outcomes in patients presenting with locally advanced disease despite refinements in surgical, radiotherapeutic, and systemic treatment strategies [1]. This unfavorable trajectory is only partially explained by classical clinicopathological parameters such as tumor size, histological grade, or nodal status, suggesting that the biological behavior of HNSCC is shaped to a considerable extent by processes occurring within the surrounding tumor microenvironment (TME) rather than by the neo-plastic epithelium alone.

Chronic inflammation is now recognized as a central driver of HNSCC initiation and progression. Persistent exposure to tobacco, alcohol, and oncogenic viruses sustains a state of low-grade, unresolved inflammation within the mucosa, which remodels the sur-rounding stroma and creates a permissive niche for malignant transformation. The TME of HNSCC is a heterogeneous compartment comprising cancer-associated fibroblasts, infiltrating immune and inflammatory cells, and extracellular matrix components that jointly regulate apoptosis evasion, angiogenesis, immune escape, and invasive growth [2]. Understanding how these compartments interact has become a prerequisite for identifying biomarkers capable of refining prognosis beyond conventional staging.

Among the cellular effectors shaped by this inflammatory milieu, Langerhans cells occupy a fundamental position as the resident antigen-presenting sentinels of the squamous epithelium. Their density, assessed immunohistochemically through S100 and, more specifically, CD1a, reflects the intensity of local immune surveillance. A growing body of evidence indicates that more advanced HNSCC is associated with reduced density of these dendritic sentinels, particularly at the tumor–stroma interface, consistent with a broader pattern of immune evasion within the inflamed microenvironment [3]. This reduction in epithelial immune-sentinel density has been reported alongside, rather than independently of, the proliferative, adhesive, and vascular changes observed in the tumor itself.

Langerhans cells were selected as the central immune-cell population because they are specialized dendritic cells resident within stratified squamous epithelium, where they contribute to local antigen capture and immune surveillance [3]. Their epithelial localization is particularly relevant in HNSCC and allows their assessment in routine tissue sections using established markers such as CD1a and S100 [3]. In parallel with the loss of immune surveillance, the neoplastic epithelium undergoes progressive architectural disorganization. Loss of E-cadherin (ECAD)-mediated intercellular adhesion is a hallmark of the epithelial-to-mesenchymal transition and has been repeatedly correlated with increased proliferative activity, as reflected by the Ki-67 index, as well as with more advanced tumor stage and nodal involvement in HNSCC [4]. Similar associations have been reported at the invasive tumor front, where the reciprocal expression of epithelial and mesenchymal cadherins delineates the leading edge of local invasion [5]. These epithelial changes rarely occur in isolation, and their relationship with the surrounding vascular and lymphatic compartments remains incompletely characterized.

Angiogenesis and lymphangiogenesis represent two further, closely interrelated hallmarks of HNSCC progression. Vascular endothelial growth factor (VEGF) is the principal driver of tumor neovascularization and has been consistently associated with advanced clinical stage and adverse outcome across HNSCC subsites [6], prompting continued interest in anti-angiogenic strategies despite their still-limited clinical benefit to date [7]. Beyond blood vessel formation, double immunostaining for Ki-67 and the lymphatic endothelial marker D2-40 (podoplanin) allows the proliferative activity of lymphatic vessels to be distinguished from mere lymphatic vessel density, and has been shown to correlate with histopathological grade, and has been proposed as a readout of lymphangiogenic activity at the tumor front and in the peritumoral stroma [8].

Despite the individual prognostic relevance of immune, proliferative, adhesive, and vascular markers, they are seldom examined within a single integrative model, and their relationship to formal tumor staging is rarely addressed jointly with cross-sectional imaging. Radiological assessment, particularly of tumor volume and nodal characteristics such as extranodal extension, has become an increasingly integral component of head and neck cancer staging, complementing histopathological evaluation and informing treatment planning [9]. Examining the relationship between this immunohistochemical immune–vascular marker pattern and imaging-based staging parameters may therefore provide additional insight into the biological characteristics associated with tumor aggressiveness.

Despite evidence implicating dendritic-cell dysfunction, increased proliferation, loss of epithelial adhesion, angiogenesis, and lymphangiogenesis in HNSCC, these processes have largely been investigated separately. It therefore remains unclear whether reduced epithelial Langerhans-cell density is associated, within the same tumors, with a coordinated pattern of proliferative, adhesion-related, angiogenic, and lymphangiogenic changes and whether these associations vary according to tumor stage and nodal status. We hypothesized that advanced and node-positive HNSCC would exhibit lower epithelial Langerhans-cell density, assessed primarily by CD1a, together with higher proliferative activity (Ki-67), reduced E-cadherin-mediated adhesion, and higher angiogenic (VEGF) and lymphangiogenic (D2-40/Ki-67) activity. Accordingly, the aims of the present study were to characterize these immunohistochemical markers in a cohort of HNSCC specimens, examine their cross-sectional associations, and determine their relationships with tumor stage and nodal status.

## 2. Results

Fifty patients with histopathologically confirmed HNSCC met all inclusion criteria and were evaluated with the complete histological/immunohistochemical panel (H&E, S100, CD1a, Ki-67, Ki-67/D2-40 double staining, E-cadherin, and VEGF), together with cross-sectional imaging and combined histopathological/radiological staging.

### 2.1. Patient Cohort and Clinicopathological Characteristics

The mean patient age was 63.9 ± 8.7 years (range, 47–84 years), with a marked male predominance (43/50, 86%), consistent with the sex distribution reported in most contemporary HNSCC series. The larynx was the predominant primary site, comprising supraglottic (21/50, 42%), glottic (16/50, 32%), and transglottic (10/50, 20%) tumors, with hypopharyngeal tumors accounting for the remaining cases (3/50, 6%). Most patients were current or former smokers (44/50, 88%). Histological grading showed a predominance of moderately differentiated tumors (G2, 44%), followed by equal proportions of well- and poorly differentiated tumors (G1 and G3, 28% each). According to combined histopathological and radiological staging, the cohort was weighted toward locally advanced disease: 36/50 tumors (72%) were classified as T3–T4, 34/50 (68%) had regional nodal involvement (N+), and 40/50 (80%) presented with overall stage III–IV disease at diagnosis. The full clinicopathological profile of the cohort is summarized in Table 1.

### 2.2. Immunohistochemical Marker Expression

CD1a+ epithelial density, the primary Langerhans cell metric (see Section 4.4), ranged widely across the cohort (median 25.7 cells/mm^2^; IQR, 16.7–31.2), with 19/50 tumors (38%) classified in the low-density strata (score 0–1), indicating a notable degree of epithelial immune-sentinel reduction in this series, in keeping with previous reports of Langerhans cell/dendritic-cell rarefaction in HNSCC. S100+ density showed a concordant pattern (median 28.6 cells/mm^2^; IQR, 21.7–36.8) and is reported as a supportive marker. The composite S100/CD1a metric (median 27.6 cells/mm^2^; IQR, 19.4–33.5; 14/50 tumors [28%] low-density) was retained as a secondary sensitivity measure (see Section 2.5). The Ki-67 proliferation index was correspondingly high (median 48.0%; IQR, 39.3–59.5%), and the Ki-67/D2-40-derived pLVD, reported as an unvalidated descriptive measure only, had a median of 1.83 proliferating lymphatic vessels/mm^2^ (IQR, 1.07–2.77). E-cadherin expression was largely preserved by H-score (median 174.5; IQR, 143.0–216.5), with frank E-cadherin loss (H-score < 100) identified in only 2/50 cases (4%). High VEGF expression (IRS ≥ 4) was observed in 32/50 tumors (64%), a proportion at the upper end of the 40–75% range typically reported for VEGF positivity in HNSCC. The full distribution of marker scores is presented in Table 2.

**Figure 1:**
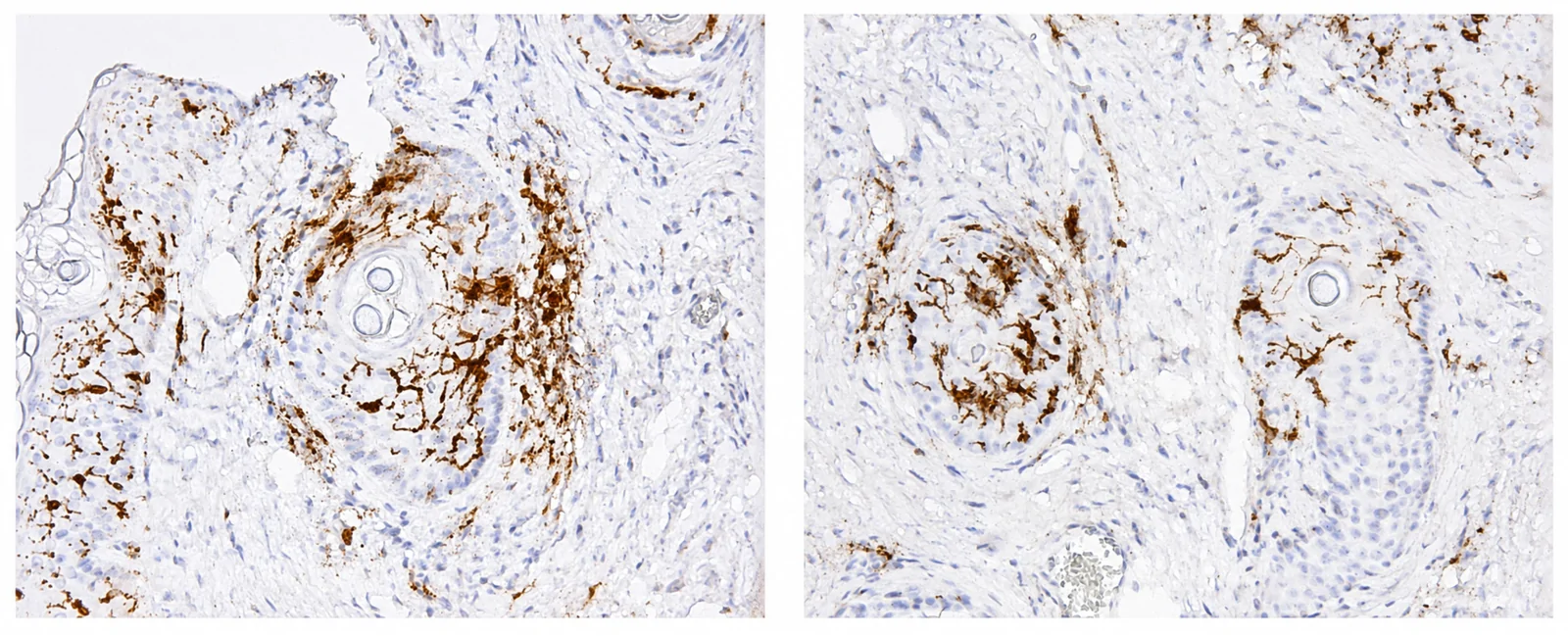
CD1a immunohistochemistry in two supraglottic laryngeal squamous cell carcinomas from the study cohort, showing CD1a+ dendritic Langerhans cells (brown) within the malignant epithelium and concentrated around keratin pearls (**left** and **right**). Original magnification 20×; scale bar: 0.050 mm.

**Figure 2:**
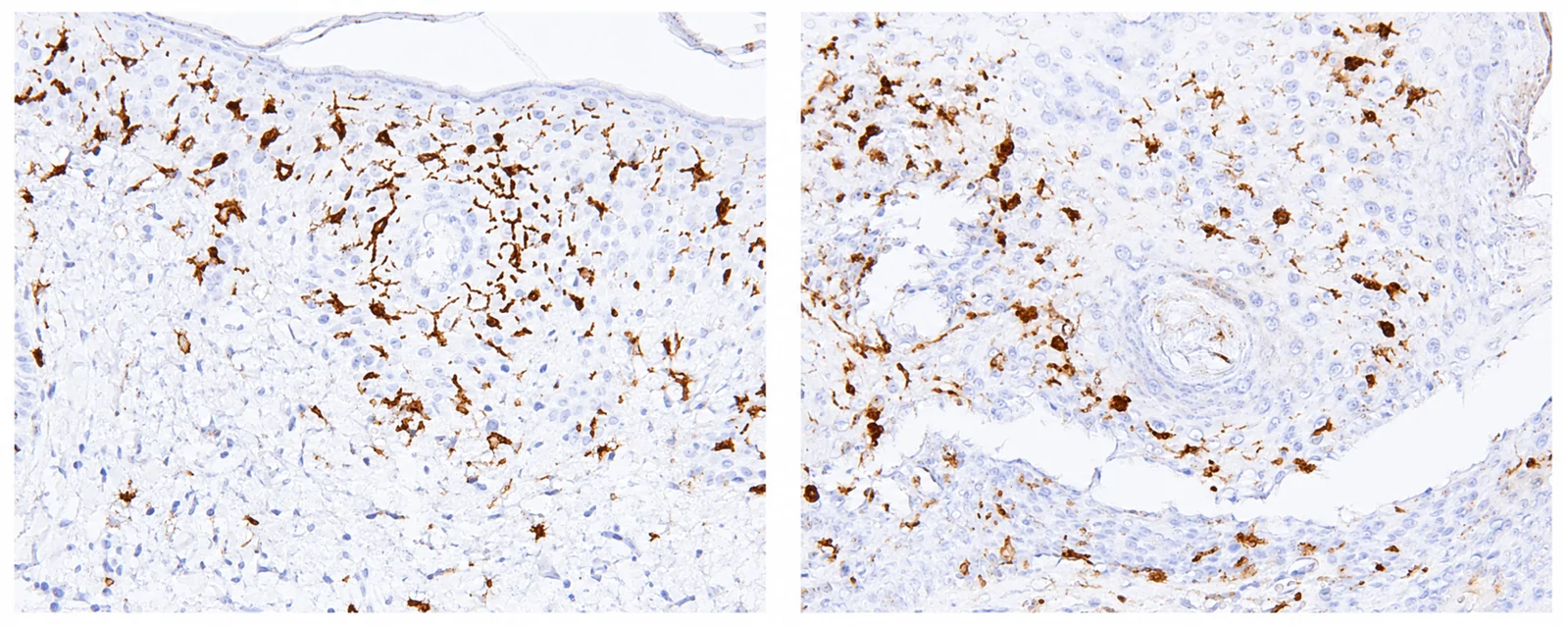
S100 immunohistochemistry in a study-cohort tumor, showing S100+ dendritic cells (brown) within the malignant epithelium, including around a keratin pearl (**right** and **left**), supporting concordance between the two Langerhans-cell markers. Original magnification 20×; scale bar: 0.050 mm.

**Figure 3:**
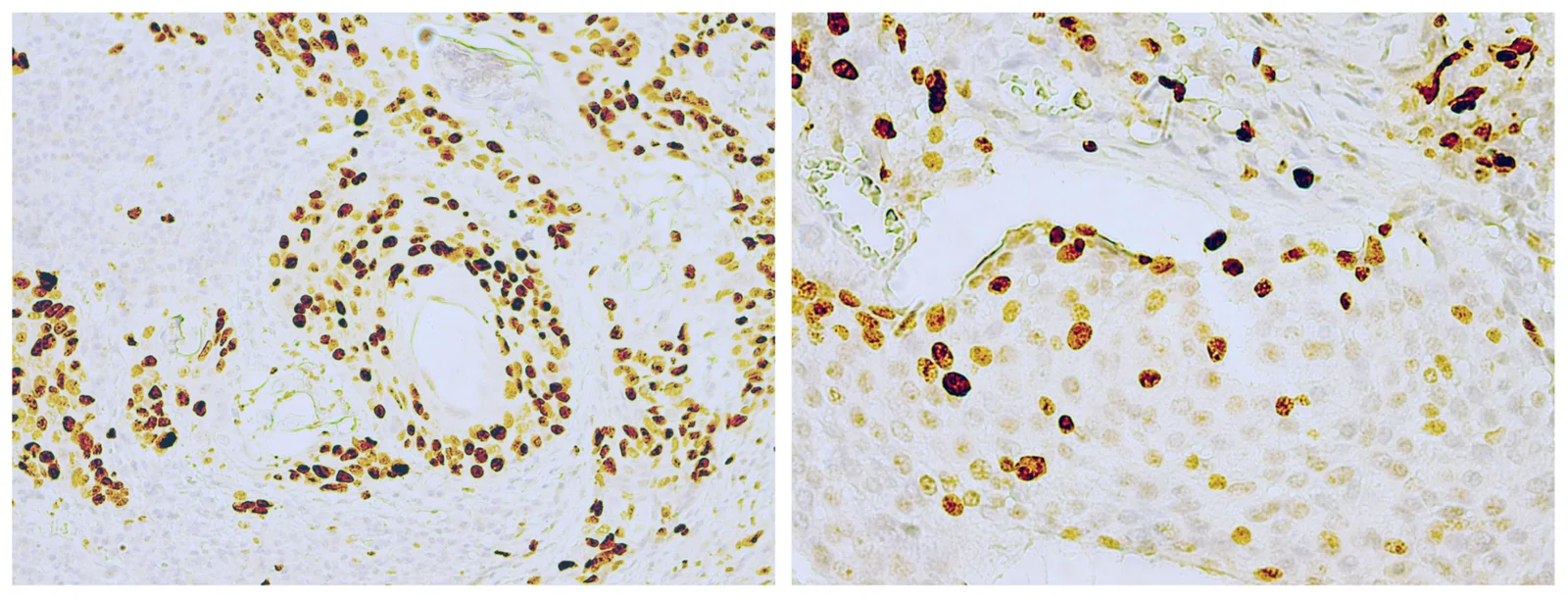
Ki-67 immunohistochemistry in two study-cohort tumors, showing nuclear positivity (dark red/brown) in a densely proliferative tumor nest (**left**, original magnification 20×; scale bar: 0.050 mm) compared with a field with a lower proliferative fraction from a different tumor, shown at higher magnification (**right**, original magnification 40×; scale bar: 0.020 mm) to better resolve individual nuclei.

**Figure 4:**
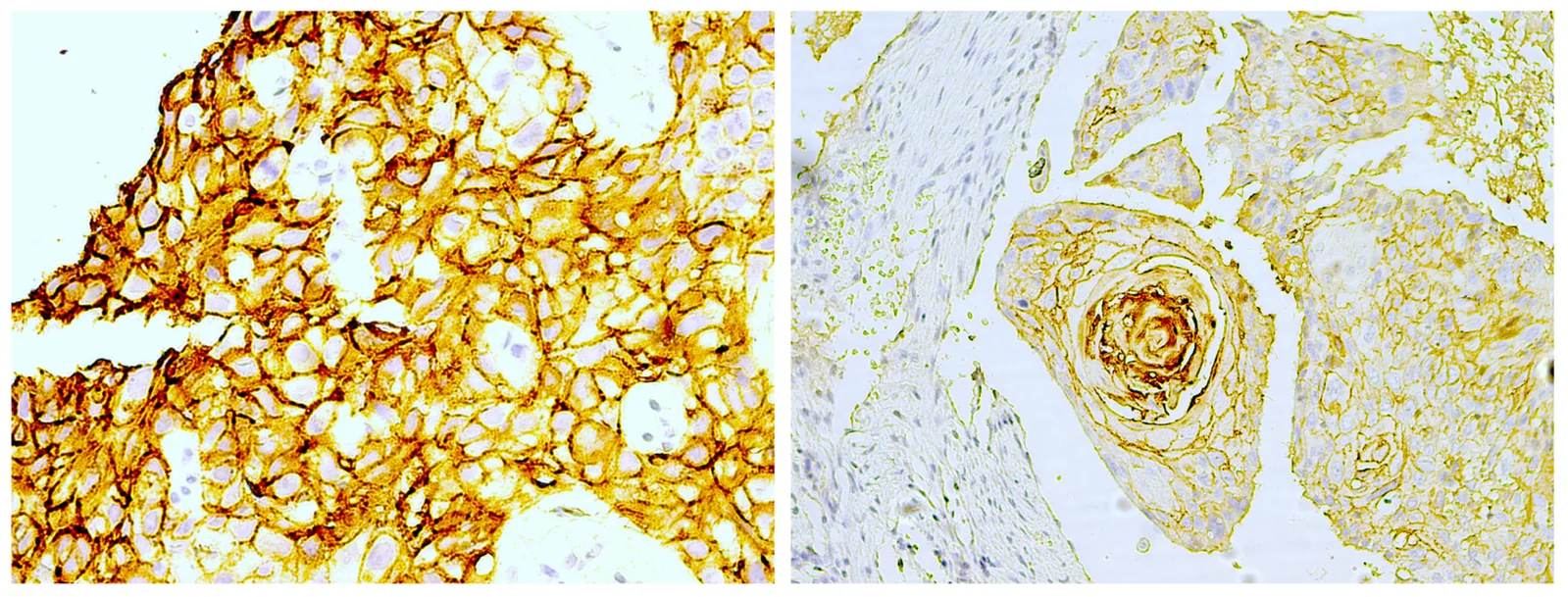
E-cadherin immunohistochemistry in two study-cohort tumors, showing strong membranous staining outlining tumor cell junctions (**left**, original magnification 40×; scale bar: 0.020 mm), and a lower-power field showing a tumor nest with central keratin pearl formation and more heterogeneous membranous positivity amid surrounding stroma (**right**, original magnification 20×; scale bar: 0.050 mm).

**Figure 5:**
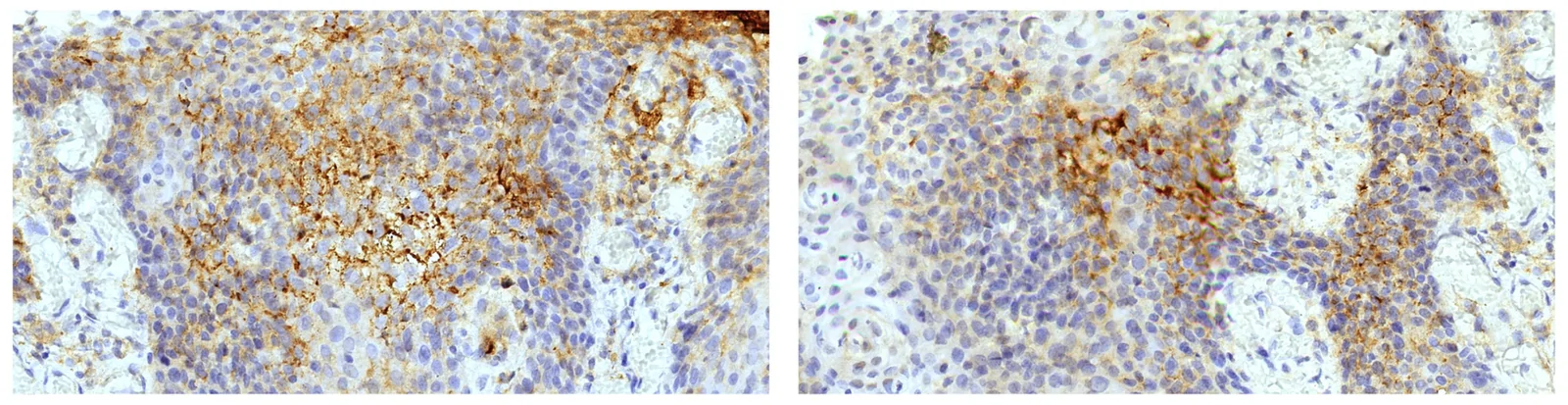
VEGF immunohistochemistry in study-cohort tumors, showing cytoplasmic immunoreactivity within tumor nests (**left** and **right**). Original magnification 20×; scale bar: 0.050 mm.

**Figure 6:**
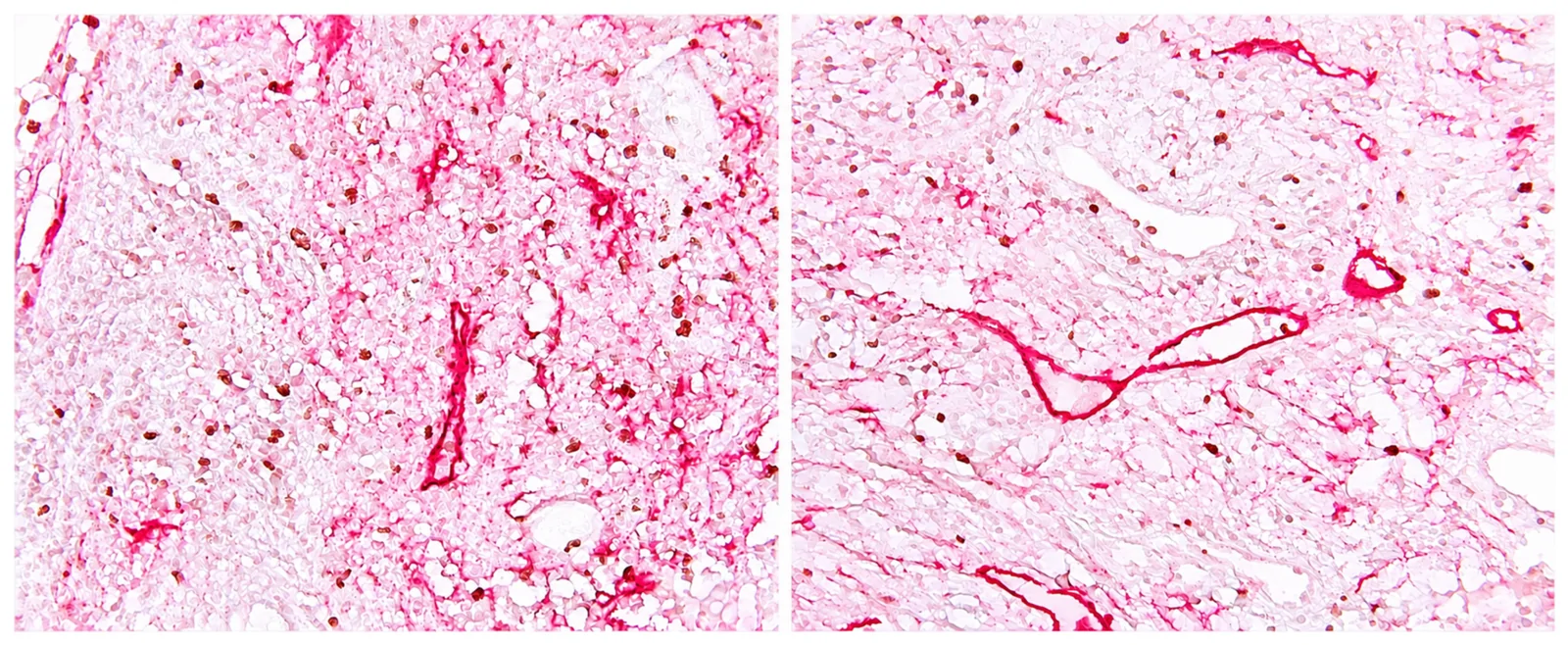
Sequential double immunohistochemistry for podoplanin (D2-40; red, Fast Red) and Ki-67 (brown, DAB) in the peritumoral stroma of two study-cohort tumors (**left** and **right**). D2-40-positive lymphatic vessels are shown together with Ki-67-positive nuclei in the surrounding stroma. At this magnification, the images do not establish endothelial nuclear localization of Ki-67 within the lymphatic vessel wall and do not distinguish endothelial from adjacent stromal or inflammatory nuclei; accordingly, lymphatic vessel density and the derived pLVD measure are reported as descriptive observations only (see Section 3 and Section 4.4.3). Original magnification 20×; scale bar: 0.020 mm.

Representative immunohistochemical staining for each of the markers evaluated is shown in Figure 1, Figure 2, Figure 3, Figure 4, Figure 5 and Figure 6. CD1a and S100 (Figure 1 and Figure 2) highlight dendritic Langerhans cells within the malignant epithelium, including around keratin pearls, corresponding to the epithelial/invasive-front compartment used for quantification. Ki-67 (Figure 3) shows nuclear positivity in proliferating tumor cells, illustrating both a densely proliferative field and a field with a lower proliferative fraction. E-cadherin (Figure 4) shows membranous staining outlining tumor cell junctions, ranging from strong and continuous to more heterogeneous positivity. VEGF (Figure 5) shows cytoplasmic immunoreactivity within tumor nests. Sequential double immunohistochemistry for D2-40 and Ki-67 is shown in Figure 6; these images do not document endothelial nuclear localization of Ki-67, and the derived pLVD measure is therefore reported descriptively only.

### 2.3. Correlations Among Immune, Proliferative, and Vascular Markers

**Figure 7:**
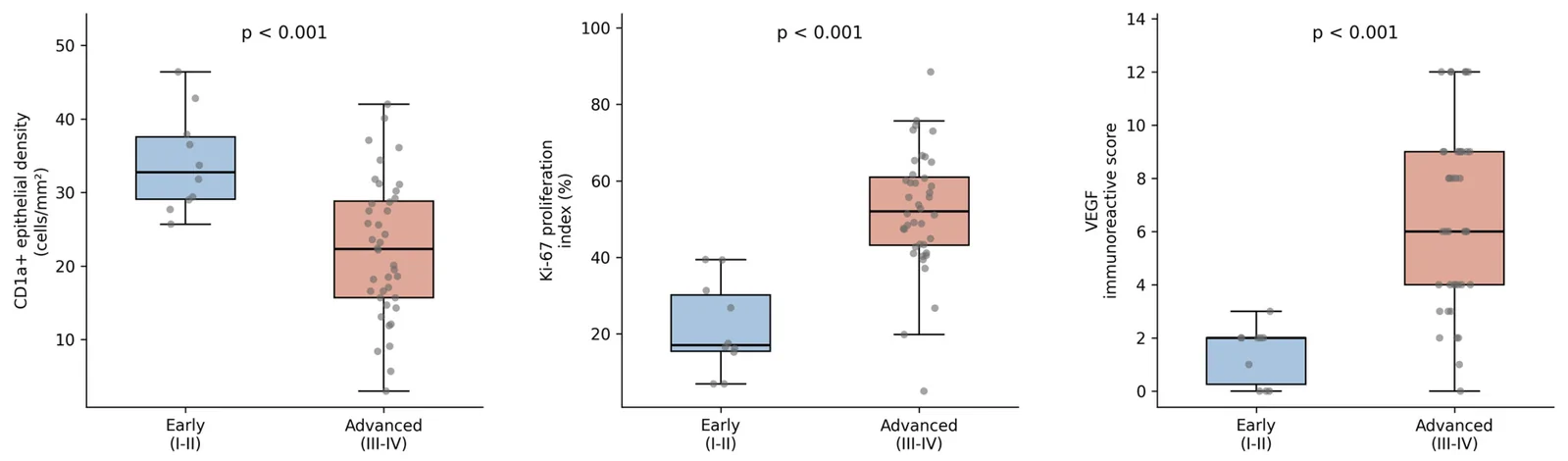
Comparison of selected immune–vascular markers between early- (stage I–II) and advanced-stage (III–IV) tumors. CD1a+ epithelial density was lower, while the Ki-67 proliferation index and VEGF immunoreactive score were higher, in advanced-stage disease (Mann–Whitney U test). Boxes show the median and interquartile range; whiskers indicate the full data range; individual points represent single cases.

CD1a+ epithelial density was significantly lower in advanced-stage (III–IV) than in early-stage (I–II) tumors (median 22.3 vs. 32.75 cells/mm^2^; *p* < 0.001), while the Ki-67 proliferation index and VEGF IRS were correspondingly higher in advanced disease (both *p* < 0.001), indicating that, in this cross-sectional series, advanced-stage tumors showed a more proliferative, VEGF-high and CD1a-low marker profile than early-stage tumors (Figure 7).

**Figure 8:**
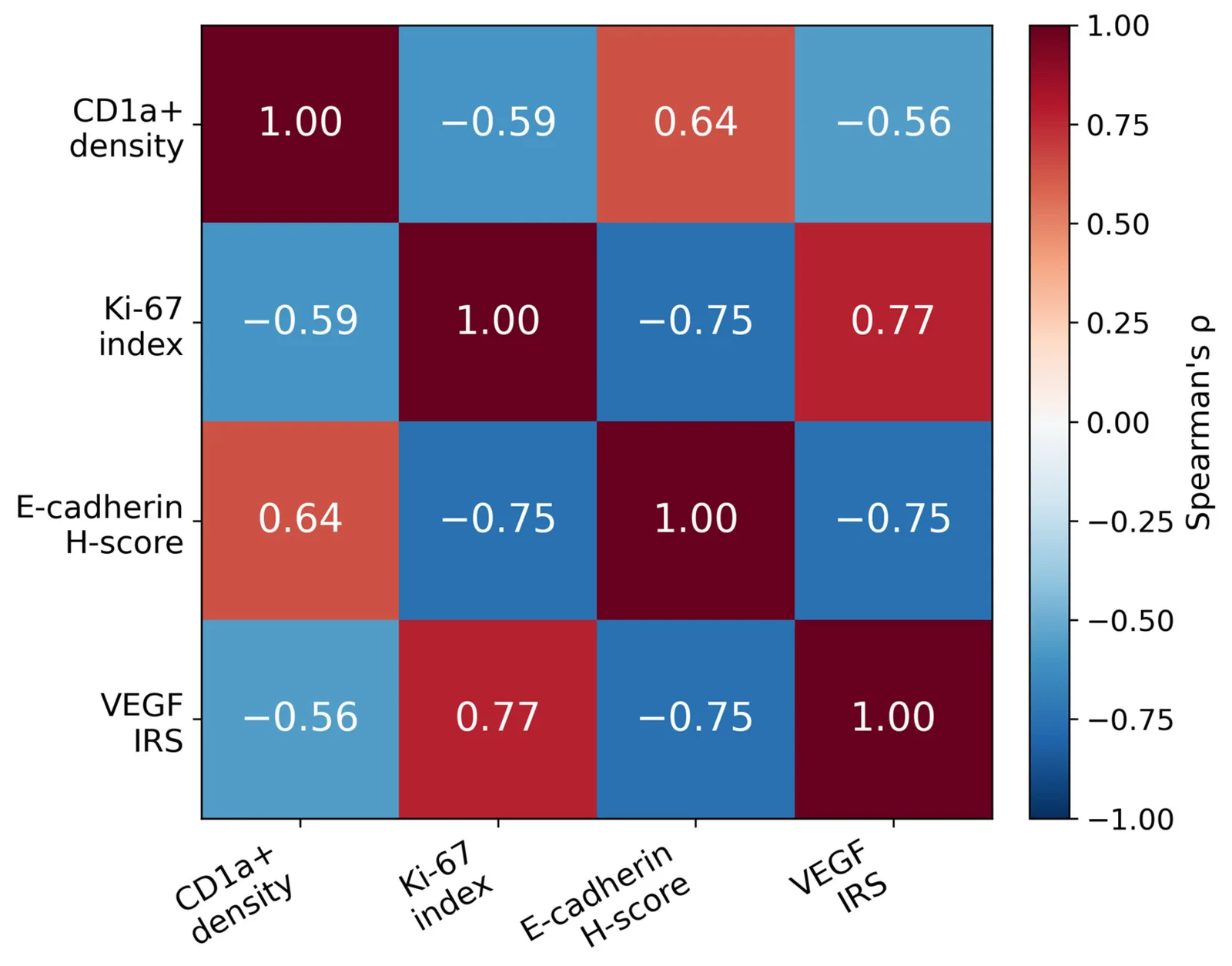
Spearman correlation matrix (ρ) among the immune–vascular markers evaluated: CD1a+ epithelial density, Ki-67 proliferation index, E-cadherin H-score, and VEGF immunoreactive score (IRS). The Ki-67/D2-40-derived pLVD was excluded because it is reported as an unvalidated descriptive observation only. Warm colors indicate positive correlation, cool colors indicate negative correlation; all coefficients shown correspond to *p* < 0.001.

Spearman correlation analysis showed a consistent pattern of associations among the four variables included in the correlation matrix: CD1a+ epithelial density correlated inversely with the Ki-67 index (ρ = −0.59) and VEGF IRS (ρ = −0.56), and positively with E-cadherin H-score (ρ = 0.64; all *p* < 0.001). Conversely, VEGF IRS correlated positively with the Ki-67 index (ρ = 0.77) and inversely with E-cadherin H-score (ρ = −0.75; all *p* < 0.001). The full correlation matrix is shown in Figure 8.

### 2.4. Association Between Marker Expression and Tumor Stage or Nodal Status

Each evaluated immune–vascular marker differed significantly between early- and advanced-stage tumors and between node-negative (N0) and node-positive (N+) cases (Table 3). Nodal involvement showed the same directional pattern as overall stage, with lower Langerhans cell density, higher proliferative activity and VEGF expression, and lower E-cadherin expression in N+ tumors.

**Figure 9:**
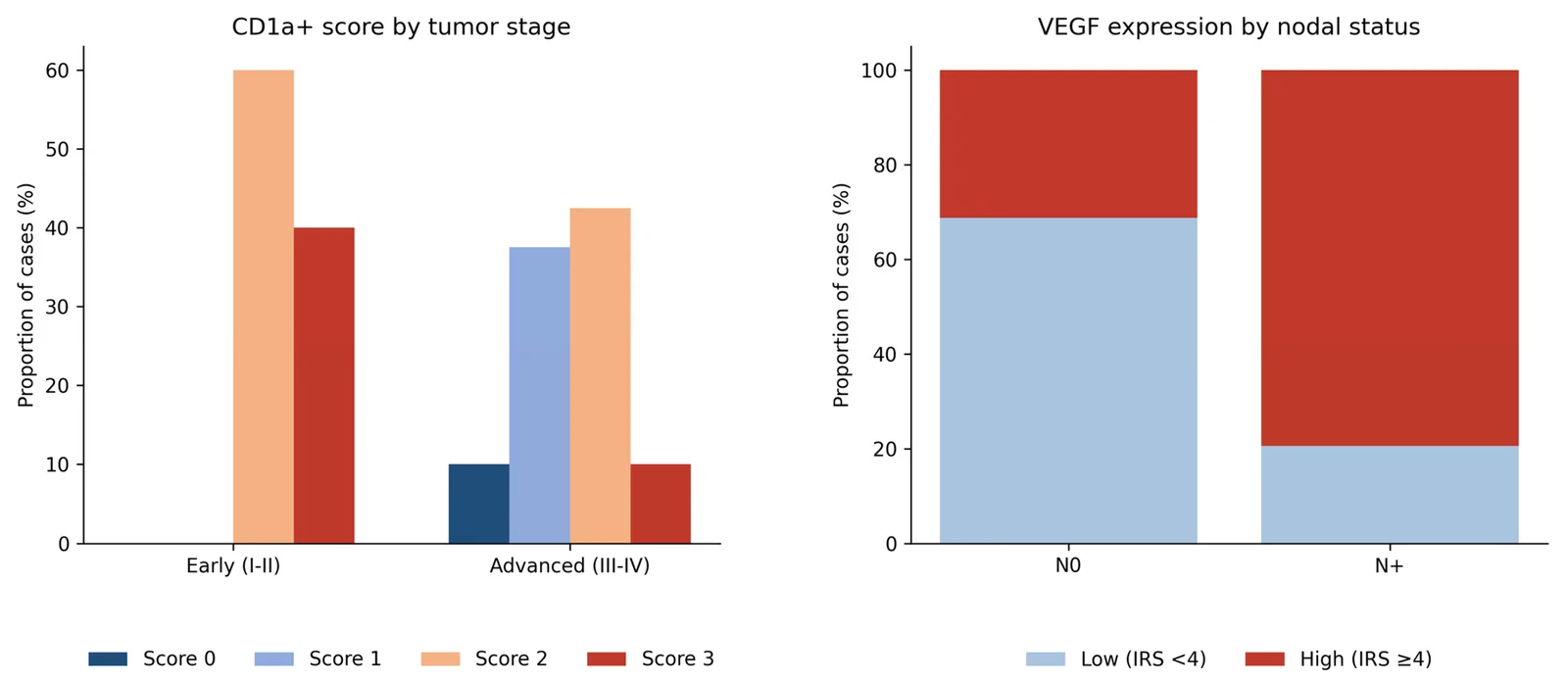
Categorical distribution of selected immune–vascular markers. (**Left**) Distribution of the CD1a+ score (0–3) by tumor stage, showing a shift toward lower scores in advanced disease. (**Right**) Proportion of low- versus high-VEGF-expressing tumors by nodal status, showing enrichment for high VEGF expression among node-positive cases.

A low CD1a score (0–1) was present in 19/40 advanced-stage tumors (48%) versus 0/10 early-stage tumors (0%); given an expected cell count below five, Fisher’s exact test was used as prespecified in Section 4.6 (*p* = 0.008). High VEGF expression (IRS ≥4) was significantly more frequent in N+ than in N0 tumors (27/34, 79% vs. 5/16, 31%; χ^2^ test, *p* = 0.003) (Figure 9).

### 2.5. Multivariable Correlates of Nodal Disease

**Figure 10:**
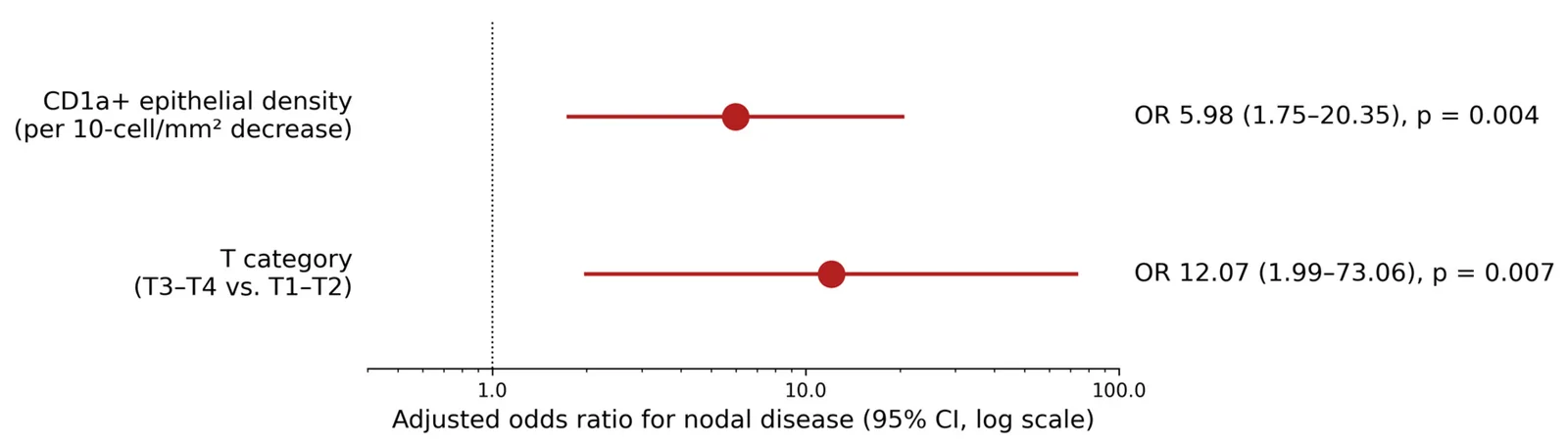
Forest plot of the multivariable logistic regression model for nodal disease (N+ vs. N0). Points represent adjusted odds ratios (OR); horizontal lines represent 95% confidence intervals; the dotted vertical line indicates OR = 1 (no effect). Red indicates statistical significance (*p* < 0.05).

A parsimonious, prespecified multivariable logistic regression model (CD1a+ epithelial density and T category) was constructed by maximum-likelihood binary logistic regression, built on 16 minority-class (N0) events and 2 model terms (EPV = 8.0), following current guidance emphasizing effective sample size and shrinkage over a fixed EPV threshold. Both CD1a+ epithelial density and T category remained associated with nodal involvement after mutual adjustment (Table 4): for every 10-cell/mm^2^ decrease in CD1a density, the odds of nodal disease increased approximately 6-fold (OR = 5.98; 95% CI, 1.75–20.35; *p* = 0.004), and T3–T4 tumors had approximately 12-fold higher odds of nodal disease than T1–T2 tumors (OR = 12.07; 95% CI, 1.99–73.06; *p* = 0.007), with both correlates showing low VIF values (VIF = 1.12) indicating no relevant collinearity (Table 4, Figure 10). The fitted model intercept was 3.97 (SE 1.75), with coefficients of −1.79 (SE 0.63) for CD1a+ density, expressed per 10-cell/mm^2^ increase (equivalent to the reported OR of 5.98 = exp(1.79) per 10-cell/mm^2^ decrease), and 2.49 (SE 0.92) for T category. The Hosmer–Lemeshow test did not indicate lack of fit (*p* = 0.463), and Nagelkerke R^2^ was 0.578; the apparent C-statistic was 0.897 and the bootstrap-corrected (optimism-adjusted, 2000 resamples) C-statistic 0.887. The apparent calibration slope is 1.00 by construction in the fitted data, whereas the bootstrap-corrected slope of 0.890 indicates some overfitting; given the small sample and the limited power of the Hosmer–Lemeshow test, these statistics do not establish good calibration, and the model was not externally validated. As a sensitivity analysis, the same model was repeated using the S100/CD1a composite metric in place of CD1a alone, yielding a concordant result (OR = 5.55 per 10-cell/mm^2^ decrease; 95% CI, 1.61–19.18; *p* = 0.007; Hosmer–Lemeshow *p* = 0.735; Nagelkerke R^2^ = 0.534), supporting the robustness of this association to marker specification. The functional form of the continuous CD1a term was assessed with a nested likelihood-ratio test comparing the primary model with a model additionally including a quadratic CD1a term (χ^2^ = 10.02, df = 1, *p* = 0.002), indicating departure from linearity on the logit scale (Table 5). Descriptively, the association was threshold-like: all 24 tumors with CD1a+ density below 25.6 cells/mm^2^ were node-positive, whereas nodal status was mixed above this value (10/26 node-positive). The odds ratio per 10-cell/mm^2^ decrease should therefore be interpreted as a linear summary of a monotonic but non-linear relationship rather than as a constant effect across the range of CD1a density; because of quasi-complete separation in the low-density range, more flexible or categorical estimates were not considered stable in this sample.

Model intercept = 3.97 (SE 1.75); coefficients: CD1a+ density = −1.79 (SE 0.63) per 10-cell/mm^2^ increase (the OR in the table is expressed per 10-cell/mm^2^ decrease, i.e., exp(1.79) = 5.98), T category = 2.49 (SE 0.92). Nagelkerke R^2^ = 0.578; Hosmer–Lemeshow goodness-of-fit (10 risk-based groups) χ^2^ = 7.71, df = 8, *p* = 0.463; apparent C-statistic = 0.897; bootstrap-corrected (optimism-adjusted, 2000 resamples) C-statistic = 0.887; apparent calibration slope = 1.00, bootstrap-corrected calibration slope = 0.890 (B = 2000; seed 20260926); events per variable (EPV) = 8.0. As a sensitivity analysis using the S100/CD1a composite metric in place of CD1a+ density alone: OR = 5.55 (95% CI, 1.61–19.18; *p* = 0.007) for the composite, OR = 7.31 (95% CI, 1.37–39.16; *p* = 0.020) for T category; Nagelkerke R^2^ = 0.534; Hosmer–Lemeshow *p* = 0.735.

As a further sensitivity analysis addressing the mixed-ascertainment nodal-status endpoint (pN for 33 surgically treated patients, imaging-based cN for 17 non-surgically treated patients), the primary model was repeated restricted to the pathologically confirmed subgroup (n = 33; 13 minority-class events, EPV = 6.5). Both correlates remained significantly associated with nodal involvement, with a consistent direction and larger point estimates (CD1a+ density: OR = 7.45 per 10-cell/mm^2^ decrease; 95% CI, 1.24–44.57; *p* = 0.028; T category: OR = 22.60; 95% CI, 1.98–257.41; *p* = 0.012), supporting the robustness of the primary findings to nodal-ascertainment method, albeit with wider confidence intervals reflecting the smaller subgroup size. A parallel imaging-only (cN) analysis across the full cohort was not feasible, as pretreatment cN was not retained for the surgically treated subgroup; this endpoint is therefore described throughout as a mixed-ascertainment nodal-status variable. Because nodal status was ascertained pathologically in surgically treated patients and clinically/radiologically in the remaining patients, and because the design is cross-sectional, the reported associations are adjusted cross-sectional associations that may be affected by differential verification and selection bias; the pN-only analysis supports directional consistency but cannot exclude these biases.

## 3. Discussion

In this cross-sectional cohort, lower epithelial Langerhans cell density was associated with higher tumor cell proliferation, lower E-cadherin expression, and higher VEGF expression, and this marker pattern was associated with both stage and mixed-ascertainment (pN/cN) nodal status. In multivariable analysis, lower CD1a+ epithelial density remained independently associated with nodal disease after adjustment for T category; given the cross-sectional design and the mixed pathological/clinical ascertainment of nodal status, this association is consistent with, though it does not establish, a role for epithelial immune-sentinel loss in advanced disease, in keeping with the persistently high global burden of this malignancy [10].

The inverse relationship observed between Langerhans cell density and tumor stage is consistent with a growing body of evidence that dendritic cells, despite their central role in antigen presentation and T-cell priming, are reduced or functionally impaired in more advanced tumors [11,12]. Chronic inflammatory signaling—sustained by tobacco and alcohol exposure and by the persistent wound-like state of the mucosal epithelium—is now recognized as a key upstream driver of this dendritic-cell dysfunction, in part through NF-κB-dependent production of pro-inflammatory and pro-angiogenic cytokines that both recruit immunosuppressive myeloid populations and impair dendritic-cell maturation [13]. This mechanism fits within the broader concept of tumor-promoting inflammation as an enabling characteristic of cancer, alongside immune evasion itself [14].

The lower E-cadherin expression observed in advanced-stage tumors may be relevant in this context, although EMT was not assessed directly in the present study. Beyond its structural role in maintaining epithelial cohesion, E-cadherin loss during epithelial-to-mesenchymal transition (EMT) has been shown to downregulate antigen-presenting capacity, favor an immunosuppressive cytokine milieu, and impair natural killer- and CD8+ T-cell-mediated cytotoxicity [15]. Similar associations between E-cadherin loss, higher proliferative activity, and invasive-front remodeling have been reported at the leading edge of oral squamous cell carcinoma [5], which is compatible with the hypothesis that EMT-like changes and reduced immune-sentinel density may be related; however, the present cross-sectional design cannot establish whether these changes are linked mechanistically or their directionality.

The strong correlation observed between VEGF expression and proliferative activity may be consistent with, among other mechanisms, tumor hypoxia and hypoxia-inducible factor-1α (HIF-1α) signaling, which upregulates VEGF transcription and promotes a stem-like, treatment-resistant phenotype [16]; hypoxia and HIF-1α were not assessed in the present study. In HNSCC specifically, dysregulation of the LIMD1–VHL–HIF-1α axis has been associated with elevated VEGF expression across clinical stages [17], and HIF-1α activation has additionally been linked to Notch1-dependent radioresistance [18] and to autophagy-mediated survival under mild hypoxic stress [19]. Hypoxia-driven EMT and acquisition of a cancer stem-cell-like phenotype [20] could offer one possible explanation for the co-occurrence of higher VEGF and lower E-cadherin expression in this cohort, although neither hypoxia nor EMT was measured.

The proliferating lymphatic vessel density derived from Ki-67/D2-40 double staining is reported in this study only as an unvalidated descriptive observation and was not used for inferential or mechanistic interpretation, because endothelial nuclear localization of Ki-67 was not documented with high-magnification images and the specificity of the double stain was not formally validated. Podoplanin-positive lymphatic endothelium has been linked to lymphatic metastasis in squamous malignancies of the head and neck, including a marked survival difference according to podoplanin status in nasopharyngeal carcinoma [21], and a meta-analysis in pulmonary squamous cell carcinoma identified podoplanin as a marker of aggressive, poorly differentiated disease [22]; whether proliferating lymphatic endothelium is associated with nodal disease in laryngeal/hypopharyngeal carcinoma requires validated assessment in future studies. In addition, broader reviews of tumor angiogenesis mechanisms have emphasized the close molecular interdependence of blood and lymphatic vessel growth programs within the same hypoxic, VEGF-rich microenvironment [23].

Importantly, VEGF is not merely a pro-angiogenic factor but also an active immunosuppressive signal, and transcriptomic analyses in HNSCC have shown that VEGF pathway activity correlates with immune cell infiltration patterns and prognosis [24]. This dual role may offer one possible link between the VEGF and immune marker patterns observed in the present study, although such a link was not tested here, and the pattern and is consistent with recent conceptual models describing tumor microenvironment-driven immune evasion, including stromal and hypoxia-dependent recruitment of immunosuppressive cells, as an important feature of HNSCC biology and resistance to immune checkpoint inhibition [25]. Reassessments of VEGF biology in anti-angiogenic therapy [26] and structural analyses of VEGFR-2 signaling [27] are compatible with such crosstalk, which remains to be tested directly.

The observed associations may also be relevant to future translational research. Immune checkpoint inhibition with pembrolizumab, alone or combined with chemotherapy, is established first-line therapy for recurrent or metastatic HNSCC [28], and several studies have investigated combinations of VEGF-pathway inhibition with EGFR- or immune-checkpoint-targeted agents, including pazopanib-cetuximab [29], bevacizumab-based chemotherapy [30], and ramucirumab-pembrolizumab [31]. A systematic review of angiogenesis inhibitors in HNSCC has similarly highlighted the need for improved biomarker-based stratification [32]. Plasma VEGF and tissue VEGFR-2 have been proposed as potential biomarkers in this context [33,34,35]. However, the present study did not assess treatment response and therefore cannot determine whether the marker pattern observed here identifies patients who may benefit from any specific therapeutic strategy. From a translational perspective, the correlated marker pattern observed in this study should be considered hypothesis-generating rather than clinically validated. Because the evaluated markers can be assessed in routine FFPE tumor material, their combined investigation may provide a feasible framework for future biomarker studies. However, the present analysis did not derive a patient-level composite score, establish clinically applicable thresholds, assess incremental performance beyond conventional staging, or evaluate survival or treatment-response endpoints. Prospective studies with predefined analytical criteria and independent validation cohorts are therefore required before any diagnostic, prognostic, predictive, or treatment-selection role can be proposed. The integration of histopathological and radiological staging in the present protocol reflects a broader trend toward multimodal risk stratification in HNSCC. Quantitative imaging approaches, including radiomics and, increasingly, deep-learning-based image analysis, have shown growing promise for prognostication and treatment planning [36], and extranodal extension on cross-sectional imaging is now recognized as an important, independent adverse feature that complements pathological nodal staging [9]. Future studies integrating the immune–vascular markers evaluated here with quantitative imaging biomarkers could determine whether these complementary data provide additional information beyond established clinicopathological assessment.

Several limitations of this study should be acknowledged. The cohort was drawn from a single institution and remains modest in size, without a formal a priori sample-size calculation, which limits precision and constrains the number of covariates that could be examined simultaneously. In addition, the marked male predominance of the cohort (86%) limits the ability to examine sex-specific differences and may reduce the generalizability of the findings to more sex-balanced HNSCC populations. Because overall stage incorporates nodal category by definition, the observed associations of the marker pattern with stage and with N status are not statistically independent validations of the same underlying relationship.

The retrospective design precluded systematic collection of HPV/p16 status and long-term survival and recurrence data. Consequently, the present findings are limited to cross-sectional associations with concurrent clinicopathological characteristics, including nodal status, and cannot establish prognostic or predictive value. Prospective cohorts with complete follow-up and clinically relevant endpoints are required to determine whether the observed marker pattern has prognostic significance. Furthermore, smoking status, alcohol consumption, and prior or concurrent treatment—factors known to modulate mucosal immune infiltration and Langerhans cell density independently of tumor biology—were not incorporated as covariates in the multivariable model; residual confounding from these variables cannot be excluded, and the reported associations should be interpreted as cross-sectional correlates rather than causal relationships.

This analysis differs from our prior reports on overlapping archival material in marker panel, biological question, and interpretation: the present study introduces an integrated CD1a+ epithelial Langerhans cell metric (with the S100/CD1a composite retained as a sensitivity measure) combined with E-cadherin, Ki-67, and VEGF within a single cohort, with the Ki-67/D2-40-derived measure reported descriptively only, whereas the earlier reports examined podoplanin-based lymphangiogenesis [8], E-cadherin/Ki-67 correlation [4], and S100/CD1a dendritic-cell density [37] as separate, single-panel analyses without cross-marker integration or nodal-status modeling.

Immunohistochemical scoring, although performed by two blinded observers with quantified interobserver agreement, remains semi-quantitative; digital image analysis and multiplex immunofluorescence could provide more granular, spatially resolved characterization of these immune and vascular markers in future studies. Finally, radiological and pathological staging were integrated at the protocol level but their respective contributions were not compared head-to-head, an analysis that would require a larger, purpose-designed cohort.

The sequential D2-40/Ki-67 double-immunostaining protocol used two mouse monoclonal primary antibodies without an interposed antibody-stripping or denaturation step, and single-stain controls formally excluding cross-detection between the two detection rounds were not generated for this cohort. Discrimination relied on the distinct subcellular compartments (membranous/vascular vs. nuclear) and chromogens (Fast Red vs. DAB) of the two signals; these features may reduce, but do not exclude, misattribution and do not constitute evidence of analytical specificity. The specificity of the double stain was therefore not formally validated by single-component controls in this cohort; for this reason, and because endothelial nuclear localization of Ki-67 was not documented with high-magnification images, the derived pLVD measure is reported only as an unvalidated descriptive observation and is not used for inferential or mechanistic interpretation.

In conclusion, this cross-sectional cohort showed that HNSCC cases with lower epithelial Langerhans cell density had a marker pattern characterized by higher proliferation, reduced epithelial adhesion, and higher VEGF expression. Lower CD1a+ epithelial density was also independently associated with concurrent nodal disease after adjustment for T category. These findings are exploratory and require prospective validation in independent cohorts with clinically relevant endpoints before any prognostic, predictive, or treatment-selection relevance can be established.

## 4. Materials and Methods

### 4.1. Study Design and Patient Cohort

This retrospective study was based on formalin-fixed, paraffin-embedded (FFPE) tumor specimens from patients with histologically confirmed head and neck squamous cell carcinoma (HNSCC), collected at the Otorhinolaryngology Clinic of Timișoara and processed at the Department of Histology, “Victor Babeș” University of Medicine and Pharmacy Timișoara. Specimens were drawn from a larger institutional archive of HNSCC cases collected during the same period (2019–2023) under the same ethics approval (No. 22, 4 September 2019), which has also contributed material to three previous reports from our group examining Ki-67/podoplanin-based lymphangiogenic activity [8], Ki-67/E-cadherin expression [4], and S100/CD1a-positive dendritic-cell density [37]. Linkage with these earlier cohorts was established from the original case list of the 2022 studies and verified against the primary pathology and medical records. Overall, 41 of the present 50 cases had been included in at least one earlier report (34/50 [68%] in reference [37], 33/50 [66%] in [8], and 31/50 [62%] in [4]); the remaining 9 cases are additional eligible cases from the same archive. On source-record review, the anatomical site of 14 linked cases differed from that previously reported (12 previously recorded as oropharyngeal and 2 as laryngeal), and the present classification follows the primary pathology/medical record. The histological grade of 13 linked cases, previously recorded as G3, was reclassified as G2 (n = 7) or G1 (n = 6) on histological reassessment of the representative block or of a different block/specimen from the same tumor. No numerical value was transferred unchanged from the earlier publications. CD1a, S100, Ki-67, E-cadherin and the proliferating-vessel fraction were re-evaluated in the present study with the protocols described in Section 4.4, on newly cut sections or on archived slides from the earlier studies; which of these applied to each case was not documented. For cases included in reference [8], D2-40 lymphatic vessel density was recalculated from the vessel counts recorded in that study and expressed as vessels/mm^2^. A case-level reconciliation table is provided as confidential Supplementary Material for editorial review. Of an initial pool of 67 tumor specimens screened for the present study, 50 histopathologically confirmed HNSCC cases fulfilled all inclusion criteria; the 17 excluded cases lacked sufficient residual tumor tissue (n = 8), had incomplete clinicopathological or staging records (n = 5), or lacked representative tumor areas suitable for the full immunohistochemical panel (n = 4). Inclusion criteria were: (1) histopathologically confirmed HNSCC; (2) adequate FFPE primary-tumor tissue for the complete histological and immunohistochemical panel (H&E, S100, CD1a, Ki-67, Ki-67/D2-40, E-cadherin, and VEGF); (3) available pretreatment cross-sectional imaging (CT and/or MRI); and (4) complete clinicopathological and staging records. Cases were excluded if residual tumor tissue was insufficient, clinicopathological or staging information was incomplete, or no representative tumor area was available for the complete immunohistochemical panel. For each patient, demographic data (age and sex), smoking status, primary tumor site, treatment category, and TNM/stage variables relevant to the present analysis were retrieved from institutional records. The final analytic cohort comprised exclusively laryngeal (supraglottic, glottic, transglottic) and hypopharyngeal primary tumors (Table 1); no oral cavity or oropharyngeal cases met inclusion criteria, and p16/HPV-dependent AJCC staging is therefore not applicable to this cohort. Anatomical subsite was tested as a candidate variable (Table 4) but was not retained in the multivariable model given the absence of association.

All immunohistochemical material was obtained from the primary tumor prior to any oncologic treatment: specimens from the 33 surgically treated patients were derived from the resected primary tumor; specimens from the 17 non-surgically treated patients were derived from the pretreatment diagnostic biopsy of the primary tumor. All pretreatment cross-sectional imaging (CT and/or MRI) used for staging was performed prior to any surgical intervention. The interval between imaging and tissue sampling was not systematically retained as a discrete variable. Seven of 50 patients (14%) presented with distant metastatic disease (M1) at diagnosis, predominantly among those receiving palliative systemic therapy (6/8); these patients were retained in the primary analysis, with nodal-assessment method and metastatic status reported transparently in Table 6.

### 4.2. Histopathological Evaluation

Representative tumor blocks were selected on the basis of hematoxylin and eosin (H&E)-stained sections by a pathologist blinded to the immunohistochemical results. Histological grading (G1–G3) was assigned according to WHO criteria and served as the principal histopathological variable used in the present analyses.

### 4.3. Immunohistochemistry

Serial 3–4-µm sections were cut from each FFPE block and immunostained using the BOND-MAX automated immunostaining platform (Leica Biosystems, Newcastle upon Tyne, UK). For single-marker immunohistochemistry, heat-induced epitope retrieval was performed using BOND Epitope Retrieval Solution 2 (Leica Biosystems), followed by incubation with the respective primary antibody. Ki-67 was detected using a mouse monoclonal antibody, clone MM1, BOND Ready-to-Use Primary Antibody (Leica Biosystems; catalog no. PA0118), with antigen retrieval for 20 min and primary-antibody incubation for 30 min at room temperature. E-cadherin was detected using a mouse monoclonal antibody, clone 36B5, BOND Ready-to-Use Primary Antibody (Leica Biosystems; catalog no. PA0387), using the same retrieval conditions and a 30-min primary-antibody incubation. CD1a was detected using a mouse monoclonal antibody, clone MTB1, BOND Ready-to-Use Primary Antibody (Leica Biosystems; catalog no. PA0235), with antigen retrieval for 20 min and primary-antibody incubation for 30 min at room temperature. S100 was detected using a polyclonal anti-S100 antibody, BOND Ready-to-Use Primary Antibody (Leica Biosystems; catalog no. PA0031), the same antibody used in our previous report [37], with antigen retrieval for 20 min and primary-antibody incubation for 30 min at room temperature. VEGF immunostaining was performed using a mouse monoclonal anti-VEGF antibody, clone VG1, processed using the same BOND automated staining platform and Polymer Refine Detection System (Leica Biosystems) as the other single-marker assays; the exact reagent catalog number and working dilution were not retained in the available laboratory records and are noted here as a limitation. For all single-marker assays, immunoreactivity was visualized using the BOND Polymer Refine Detection System (Leica Biosystems), with 3,3′-diaminobenzidine (DAB) as chromogen and hematoxylin counterstaining. Reagent lot numbers were recorded in the laboratory batch logs for individual staining runs and are available from the corresponding author upon reasonable request.

For assessment of lymphatic vessel density and proliferative activity within lymphatic endothelium, sequential double immunostaining for D2-40/podoplanin (mouse monoclonal antibody, clone D2-40, FLEX Ready-to-Use; Agilent Technologies Denmark ApS, Glostrup, Denmark; catalog no. IR072) and Ki-67 (mouse monoclonal antibody, clone MIB-1, FLEX Ready-to-Use; Agilent Technologies Denmark ApS; catalog no. IR626) was performed on the same tissue section, consistent with our previously described approach [8]. Heat-induced epitope retrieval was performed using BOND Epitope Retrieval Solution 2 for 20 min, followed by endogenous peroxidase blocking for 5 min. Sections were incubated with the D2-40 primary antibody for 20 min and visualized using the BOND Polymer Refine Red Detection System with Fast Red chromogen, producing a red-purple signal. Sequential incubation with the Ki-67 primary antibody for 20 min was followed by visualization using the BOND Polymer Refine Detection System with DAB, producing a brown nuclear signal. Hematoxylin was used as counterstain. This sequential double-chromogen protocol relied on the distinct subcellular localization of the two antigens (D2-40: membranous/cytoplasmic, endothelial; Ki-67: nuclear) and their distinct chromogens (Fast Red for D2-40, DAB for Ki-67) to discriminate D2-40-positive lymphatic endothelial profiles from Ki-67-positive proliferating nuclei. No antibody-stripping or denaturation step was performed between the two primary-antibody incubations, and no single-stain controls were generated to formally exclude carryover cross-reactivity between the two mouse monoclonal antibodies. The distinct chromogens and subcellular localizations therefore do not by themselves establish analytical specificity, and the specificity of the double stain was not formally validated in this cohort; this is acknowledged as a methodological limitation (Section 3), and the derived pLVD measure is accordingly reported descriptively only.

No separate blood-vessel endothelial immunostain (e.g., CD31 or CD34) was used to validate blood-vessel identity. We therefore do not report a validated microvessel density (MVD). Instead, a morphology-based vascular-profile count was recorded manually by light microscopy in the same D2-40-stained hotspot fields used for lymphatic-vessel evaluation, expressed as the total number of morphologically identifiable vascular profiles per mm^2^, including both D2-40-positive and D2-40-negative luminal profiles; D2-40-positive profiles were additionally recorded separately as lymphatic vessel density. This count, reported in Table 2 and in the “Morphology_based_vascular_profile_count_vessels_mm2” column of the Supplementary Dataset, therefore represents all vascular profiles and was not confirmed with a blood-endothelial marker. Vessel counts were performed directly by the observers at high magnification; no automated image-analysis or digital vessel-counting software was used. Because this count lacks marker-based validation of blood-vessel identity, it is reported descriptively only (Table 2) and was excluded from the correlation matrix (Figure 8), the stage/nodal comparison (Table 3), and the univariate regression screen (Table 4).

Sections processed without primary antibody served as negative reagent controls. Human tonsil tissue was used as positive-control tissue for the immunohistochemical panel. Germinal-center lymphocytes provided a positive control for Ki-67; stratified squamous epithelium with preserved membranous staining served as a control for E-cadherin; Langerhans cells within squamous epithelium provided positive-control elements for S100 and CD1a; and native subepithelial vascular structures provided positive-control elements for D2-40. For the D2-40/Ki-67 double stain, native D2-40-positive lymphatic vessels and proliferating lymphoid cells provided the respective positive-control elements. Appropriate VEGF-positive tissue structures were evaluated according to the previously established VEGF staining protocol.

### 4.4. Immunohistochemical Scoring

All slides were evaluated independently by two observers blinded to clinical and staging data; discrepancies were resolved by joint review at a multi-head microscope. Interobserver agreement was quantified using weighted Cohen’s κ for ordinal scores and the intraclass correlation coefficient (ICC, two-way random-effects, absolute agreement) for continuous densities, with κ ≥ 0.61 and ICC ≥ 0.75 considered indicative of substantial/excellent agreement, respectively. Marker-specific estimates with 95% confidence intervals are reported in Table 7. Interobserver agreement for immunohistochemical scoring. Formal agreement statistics (κ/ICC) were not computed for the proliferating fraction, pLVD, or continuous VEGF IRS, as paired pre-reconciliation observer values were not retained separately for these derived/continuous measures; all measures were nonetheless scored under the same dual-observer protocol described above, with discrepancies resolved by joint review.

#### 4.4.1. S100 and CD1a

CD1a+ and S100+ dendritic/Langerhans-type cells were counted in the present study separately in the tumor epithelium/invasive front and in the peritumoral stroma, in five non-overlapping high-power fields (400×; field area 0.238 mm^2^) selected in the areas of highest density, and densities were expressed as cells/mm^2^ (mean of the five fields). For the 16 cases not included in reference [37], counts were performed on newly stained sections; for the 34 overlapping cases, counts were performed on newly cut and stained sections or on the archived S100/CD1a slides from that study. All counts were newly performed with the present protocol; no values were re-scored from images, recalculated from earlier data, or taken from the previous publication. This protocol differs from that of our previous report [37], in which S100+ and CD1a+ cells were counted in four high-power fields in the intratumoral and peritumoral areas of highest density and expressed as an ordinal score (0, negative; +1, 1–20%; +2, 21–50%; +3, 51–100% dendritic cells). The previous ordinal scores and the present continuous densities are therefore not interchangeable, and the study-specific 0–3 density strata described below are not equivalent to the 0–3 score used in [37]. Densities were additionally stratified on an ordinal 0–3 scale (<10, 10 < 22, 22 < 36, ≥36 cells/mm^2^) to model the degree of immune-sentinel depletion. The four density strata were study-specific descriptive categories used to facilitate presentation of the distribution of dendritic/Langerhans cell density; they were not derived as validated clinical thresholds and were not used as the primary continuous exposure in inferential modeling. Primary association and regression analyses were performed using continuous CD1a+ epithelial density. Because S100 lacks specificity for Langerhans cells, interpretation relied primarily on CD1a positivity, with S100 used as a corroborative marker in the presence of concordant dendritic morphology. Epithelial/invasive-front CD1a+ density was therefore treated as the primary Langerhans cell metric for all inferential analyses; S100+ density was analyzed separately as a supportive marker, and the S100/CD1a composite (arithmetic mean of the S100+ and CD1a+ densities) was retained as a secondary sensitivity measure. Peritumoral stromal counts for both markers were quantified during scoring using the same protocol but were not incorporated into the primary Langerhans cell metric, as the primary analysis specifically targets intraepithelial/invasive-front CD1a+ cells. Stromal dendritic-cell populations are biologically distinct from epithelial Langerhans cells; these stromal counts are not reported in this article and are available from the corresponding author upon reasonable request. Patient-level mean densities (averaged across five high-power fields per marker) are available in Supplementary Dataset S1; field-level counts were not retained as a separate dataset beyond the field means recorded at the time of scoring.

#### 4.4.2. Ki-67

The proliferation index was determined as the percentage of Ki-67-positive tumor nuclei in three to five hotspot fields (400×). For descriptive presentation, values were additionally categorized on a 0–4 ordinal scale (0: <10%; 1: 10–<25%; 2: 25–<50%; 3: 50–<75%; 4: ≥75%). These categories were treated as exploratory descriptive strata rather than validated clinical thresholds; inferential analyses used the continuous Ki-67 proliferation index.

#### 4.4.3. Ki-67/D2-40 Double Staining

Lymphatic vessel density (LVD) was calculated as the number of D2-40-positive vascular profiles per mm^2^ in the tumor and peritumoral compartments. The proportion of D2-40-positive vessels containing Ki-67-positive endothelial nuclei was recorded as the percentage of proliferating lymphatics, and proliferating lymphatic vessel density (pLVD) was derived as the product of LVD (vessels/mm^2^) and the proliferating lymphatic fraction, with the proliferative proportion entered as a decimal fraction ranging from 0 to 1; the resulting derived measure is therefore expressed as proliferating lymphatic vessels/mm^2^, adapted from the double-staining approach previously described by our group [8]. Endothelial nuclear localization of Ki-67 positivity was assessed at high magnification during scoring; however, because it could not be documented with representative high-magnification images and the double-stain specificity was not formally validated (Section 3), lymphatic vessel density, the proliferating fraction and pLVD are reported only as descriptive observations (Table 2) and were excluded from the correlation matrix, the stage/nodal comparisons, the regression analyses and mechanistic interpretation.

#### 4.4.4. E-Cadherin

Membranous immunoreactivity in tumor cells was evaluated using a combined intensity–extent H-score (range 0–300), calculated as staining intensity (0–3) multiplied by the percentage of positive cells. For descriptive analyses, an H-score < 100 was categorized as reduced/lost expression, and values were additionally stratified into a 0–3 ordinal scale (0: <50; 1: 50–99; 2: 100–199; 3: ≥200). These cutoffs were treated as exploratory descriptive thresholds rather than validated clinical cutoffs; inferential analyses used the continuous H-score. Given the recognized heterogeneity of membranous staining, both the invasive front and the tumor center were sampled.

#### 4.4.5. VEGF

Cytoplasmic immunoreactivity was evaluated separately in tumor cells, stroma, and tumor-associated endothelium. An immunoreactive score (IRS, range 0–12) was calculated as the product of staining intensity (0–3) and the extent of positive cells (proportion score 0–4). For descriptive analyses, IRS 0–3 was classified as low expression and IRS ≥4 as high expression. This dichotomization was treated as exploratory and was not used as the primary continuous exposure in the multivariable model.

### 4.5. Radiological Assessment and Staging

Pretreatment contrast-enhanced CT and/or MRI studies, available for all included patients, were reviewed by a radiologist with dedicated experience in head and neck oncologic imaging, blinded to the immunohistochemical results. Primary tumor dimensions, depth of invasion where assessable, and regional lymph node characteristics (number, short-axis diameter, and imaging features suggestive of extranodal extension) were recorded. TNM categories and overall stage group were assigned according to the 8th edition AJCC staging system [9]. The nodal disease outcome used in this study followed a hierarchical definition: pathological nodal status (pN) was used for the 33/50 patients who underwent primary surgical treatment with neck dissection; pretreatment clinical/radiological nodal status (cN), based on contrast-enhanced CT/MRI, was used for the remaining 17/50 patients treated non-surgically (definitive chemoradiotherapy or palliative systemic therapy). Because paired cN and pN values were not retained as separate data fields for the surgically treated subgroup, formal radiological–pathological concordance could not be assessed in the present series.

### 4.6. Statistical Analysis

Statistical analyses were performed using SPSS software (Statistical Package for the Social Sciences; IBM Corp., Armonk, NY, USA; version 26.0). Key inferential results (the multivariable model in Section 2.5, the Spearman correlation matrix, and the surgical-subgroup sensitivity analysis) were independently cross-validated using Python (v3.x; pandas, NumPy, SciPy, statsmodels, scikit-learn), yielding concordant estimates. Continuous variables were summarized as mean ± standard deviation or median (interquartile range), depending on distribution, and categorical variables as frequencies and percentages. Associations between immunohistochemical markers (S100, CD1a, Ki-67, E-cadherin, and VEGF) and clinicopathological or staging variables (tumor stage, T category, nodal status, histological grade) were evaluated using the chi-square test or Fisher’s exact test, as appropriate. Correlations among the continuous or ordinal immunohistochemical variables were assessed using Spearman’s rank correlation coefficient, and comparisons of marker levels between two or more independent groups were performed using the Mann–Whitney U test or Kruskal–Wallis test, as appropriate.

Univariate logistic regression was used to examine candidate variables associated with nodal disease (N+ vs. N0). CD1a+ epithelial density was prespecified as the primary marker of interest, and T category was prespecified for inclusion in the multivariable model because of its established clinical relevance to nodal status. Other candidate clinicopathological and immunohistochemical variables were evaluated in univariate analyses and are reported as exploratory. Given the modest sample size, the multivariable model was restricted a priori to two terms, CD1a+ epithelial density and T category, yielding 16 minority-class (N0) observations for two model terms (EPV = 8.0). No recurrence model or penalized regression analysis was performed. Linearity of the continuous CD1a term on the logit scale was assessed with a nested likelihood-ratio test against a model including an additional quadratic CD1a term. Internal validation used non-parametric bootstrap resampling of cases with replacement (2000 resamples; random seed 20260926), with optimism correction of the C-statistic and calibration slope; resamples containing a single outcome class, failing to converge, or showing (quasi-)complete separation (any |coefficient| > 20) were discarded (38/2000), and statistics were averaged over the remaining resamples. Multicollinearity among predictors was assessed using Variance Inflation Factors (VIF), model calibration was evaluated with the Hosmer–Lemeshow goodness-of-fit test, and explanatory power was expressed as the Nagelkerke pseudo-R^2^. Results of logistic regression analyses are reported as odds ratios (OR) with 95% confidence intervals (CI). A two-tailed *p*-value < 0.05 was considered statistically significant throughout. CD1a+ epithelial density was prespecified as the primary marker of interest; all other marker correlations and group comparisons (Table 2 and Table 4, Figure 8) are reported as exploratory, without adjustment for multiple comparisons.

## 5. Conclusions

This cross-sectional study showed that lower epithelial Langerhans cell density in HNSCC was associated with higher proliferative activity, reduced E-cadherin-mediated adhesion, and higher VEGF expression. This correlated marker pattern was associated with tumor stage and nodal status, while lower CD1a+ epithelial density remained independently associated with concurrent nodal disease after adjustment for T category. The cross-sectional design does not establish temporal, causal, prognostic, or predictive relationships among these findings. Accordingly, the observed marker pattern should be regarded as exploratory and hypothesis-generating, particularly in view of the modest, single-center cohort, marked male predominance, and absence of systematic HPV/p16 and longitudinal outcome data. Prospective, multicenter studies with predefined analytical criteria, independent validation, and clinically relevant endpoints are required to determine whether these biomarkers provide information beyond established clinicopathological assessment.

## Figures and Tables

**Table 1:** Clinicopathological characteristics of the study cohort (n = 50).

Variable	Category	n (%)/Value
Age (years)	Mean ± SD	63.9 ± 8.7
	Range	47–84
Sex	Male	43 (86)
	Female	7 (14)
Primary site	Supraglottic larynx	21 (42)
	Glottic larynx	16 (32)
	Transglottic larynx	10 (20)
	Hypopharynx	3 (6)
Smoking status	Current	32 (64)
	Former	12 (24)
	Never	6 (12)
Histological grade (H&E)	G1 (well differentiated)	14 (28)
	G2 (moderately differentiated)	22 (44)
	G3 (poorly differentiated)	14 (28)
T category	T1–T2	14 (28)
	T3–T4	36 (72)
N category	N0	16 (32)
	N+ (N1–N3)	34 (68)
M category	M0	43 (86)
	M1	7 (14)
Overall stage group	Early (I–II)	10 (20)
	Advanced (III–IV)	40 (80)

**Table 2:** Distribution of immunohistochemical marker expression (n = 50). ᵃ Morphology-based count of all morphologically identifiable vascular profiles, including D2-40-positive and D2-40-negative profiles (see Section 4.3); not validated against a blood-endothelial marker (e.g., CD31/CD34), not equivalent to microvessel density, and reported descriptively only; excluded from correlation, group-comparison and regression analyses. ᵇ Measures derived from the sequential Ki-67/D2-40 double stain, for which single-stain specificity controls and high-magnification documentation of endothelial Ki-67 localization were not available; reported as descriptive observations only and excluded from all correlation, group-comparison and regression analyses.

Marker	Metric	Value
S100+ density (cells/mm^2^)	Median (IQR)	28.6 (21.7–36.8)
CD1a+ density (cells/mm^2^)	Median (IQR)	25.7 (16.7–31.2)
Composite Langerhans cell density (cells/mm^2^)	Median (IQR)	27.6 (19.4–33.5)
Langerhans cell score (0–3)	0/1/2/3	1 (2%)/13 (26%)/28 (56%)/8 (16%)
CD1a-only score (0–3)	0/1/2/3	4 (8%)/15 (30%)/23 (46%)/8 (16%)
Ki-67 proliferation index (%)	Median (IQR)	48.0 (39.3–59.5)
Ki-67 score (0–4)	0/1/2/3/4	3 (6%)/5 (10%)/20 (40%)/20 (40%)/2 (4%)
D2-40 lymphatic vessel density (vessels/mm^2^) ᵇ	Median (IQR)	12.1 (8.9–14.9)
Proliferating (Ki-67+) lymphatics (%)ᵇ	Median (IQR)	15.2 (10.6–19.6)
Proliferating lymphatic vessel density ᵇ	Median (IQR)	1.83 (1.07–2.77)
E-cadherin H-score (0–300)	Median (IQR)	174.5 (143.0–216.5)
E-cadherin loss (H-score < 100)	n (%)	2 (4%)
VEGF immunoreactive score (IRS, 0–12)	Median (IQR)	5.0 (2.0–9.0)
High VEGF expression (IRS ≥ 4)	n (%)	32 (64%)
Morphology-based vascular-profile count (vessels/mm^2^) ᵃ	Median (IQR)	29.8 (25.0–35.4)

**Table 3:** Association between immune–vascular marker levels (median, IQR) and tumor stage or nodal status (Mann–Whitney U test).

Marker	Early Stage (I–II)	Advanced Stage (III–IV)	*p*-Value	N0	N+	*p*-Value
Ki-67 index (%)	17.0 (15.4–30.2)	52.0 (43.1–60.9)	<0.001	29.1 (16.4–44.3)	53.2 (43.3–61.4)	0.001
E-cadherin H-score	250.5 (231.8–272.8)	163.5 (141.8–189.0)	<0.001	232.5 (184.5–263.0)	163.0 (141.2–187.5)	<0.001
VEGF IRS	2.0 (0.2–2.0)	6.0 (4.0–9.0)	<0.001	2.0 (1.8–4.0)	8.0 (4.0–9.0)	<0.001
CD1a+ epithelial density (cells/mm^2^)	32.75 (29.10–37.55)	22.30 (15.70–28.83)	<0.001	31.15 (28.88–34.93)	19.05 (14.95–27.08)	<0.001
Composite S100/CD1a density (cells/mm^2^)	36.0 (32.2–38.7)	24.2 (18.4–30.9)	<0.001	33.6 (28.6–39.1)	23.6 (17.9–29.9)	<0.001

**Table 4:** Multivariable logistic regression analysis of correlates of nodal disease.

Correlate	OR	95% CI	*p*-Value	VIF
CD1a+ epithelial density (per 10-cell/mm^2^ decrease)	5.98	1.75–20.35	0.004	1.12
T category (T3–T4 vs. T1–T2)	12.07	1.99–73.06	0.007	1.12

**Table 5:** Univariate logistic regression screen of all candidate variables for nodal disease (N+ vs. N0).

Candidate Variable	OR	95% CI	*p*-Value
CD1a+ epithelial density (per 10-cell/mm^2^ decrease)	5.43	1.95–15.12	0.0012
S100+ epithelial density (per 10-cell/mm^2^ decrease)	2.55	1.27–5.14	0.0087
Ki-67 proliferation index (per 10% increase)	2.01	1.30–3.12	0.0017
E-cadherin H-score (per 10-unit decrease)	1.35	1.13–1.61	0.0008
VEGF IRS (per 1-unit increase)	1.51	1.17–1.97	0.0018
Age (per 1-year increase)	0.97	0.90–1.04	0.3956
T category (T3–T4 vs. T1–T2)	12.50	2.92–53.48	0.0007
Histological grade (G3 vs. G1–G2)	2.07	0.49–8.80	0.3234
Primary site (supraglottic vs. other)	1.32	0.39–4.45	0.659

**Table 6:** Cohort stratified by method of nodal status assessment.

	Pathological (pN) Surgically Treated (n = 33)	Clinical/Radiological (cN) Non-Surgically Treated (n = 17)
T1/T2/T3/T4a/T4b	6/5/13/7/2	1/2/7/6/1
N0/N1/N2/N3	13/5/8/7	3/7/6/1
Stage I/II/III/IVA/IVB/IVC	4/5/7/8/9/0	0/1/5/3/1/7
M0/M1	33/0	10/7

**Table 7:** Interobserver agreement for immunohistochemical scoring, expressed as Cohen’s κ (ordinal and binary scores) and intraclass correlation coefficient (continuous measures), with 95% confidence intervals.

Marker/Variable	Statistic	Value	95% CI
CD1a score (0–3)	Weighted Cohen’s κ	0.81	0.69–0.90
S100 score (0–3)	Weighted Cohen’s κ	0.78	0.65–0.87
Ki-67 score (0–4)	Weighted Cohen’s κ	0.84	0.73–0.91
VEGF high/low	Cohen’s κ	0.86	0.73–0.94
E-cadherin category	Weighted Cohen’s κ	0.79	0.66–0.88
CD1a density (continuous)	ICC	0.89	0.82–0.94
S100 density (continuous)	ICC	0.86	0.77–0.92
Ki-67 proliferation index	ICC	0.92	0.87–0.96
E-cadherin H-score	ICC	0.87	0.79–0.92
D2-40/LVD	ICC	0.88	0.80–0.93

Intraclass correlation coefficient (two-way random-effects, absolute agreement). Ordinal scores (CD1a, S100, Ki-67, E-cadherin category) were assessed using weighted Cohen’s κ; the binary VEGF high/low classification was assessed using unweighted Cohen’s κ; continuous densities (cells/mm^2^ or H-score) were assessed using ICC. κ ≥ 0.61 and ICC ≥ 0.75 were considered indicative of substantial/excellent agreement, respectively.

## Data Availability

The de-identified patient-level dataset (Supplementary Dataset S1), data dictionary, and Python analysis script are provided as confidential Supplementary Material for editorial and peer-review evaluation. Access to these materials is restricted by institutional data-sharing policies. The original SPSS output files and immunohistochemical scoring sheets are not included in the submission but may be requested from the corresponding author upon reasonable request and in compliance with institutional ethics requirements.

## Supplementary Materials

The following supporting information can be downloaded at: https://www.mdpi.com/article/10.3390/ph19101564/s1. Supplementary Dataset S1: De-identified patient-level data (age, sex, smoking status, anatomical site, histological grade, TNM categories, stage, treatment category, and immunohistochemical markers); Supplementary Data Dictionary S1: Variable definitions and coding instructions; Supplementary Script S1: Python script for statistical analysis reproduction.

## Abbreviations

The following abbreviations are used in this manuscript:

AJCC	American Joint Committee on Cancer
CI	Confidence interval
CT	Computed tomography
DAB	3,3′-diaminobenzidine
ECAD	E-cadherin
EGFR	Epidermal growth factor receptor
EMT	Epithelial-to-mesenchymal transition
EPV	Events per variable
FFPE	Formalin-fixed, paraffin-embedded
HIF-1α	Hypoxia-inducible factor-1α
HNSCC	Head and neck squamous cell carcinoma
HPV	Human papillomavirus
IQR	Interquartile range
IRS	Immunoreactive score
LVD	Lymphatic vessel density
MRI	Magnetic resonance imaging
NF-κB	Nuclear factor kappa B
OR	Odds ratio
SPSS	Statistical Package for the Social Sciences
TME	Tumor microenvironment
TNM	Tumor, node, metastasis
VEGF	Vascular endothelial growth factor
VEGFR-2	Vascular endothelial growth factor receptor 2
VIF	Variance inflation factor
WHO	World Health Organization
